# General‐purpose genotypes and evolution of higher plasticity in clonality underlie knotweed invasion

**DOI:** 10.1111/nph.20452

**Published:** 2025-02-19

**Authors:** Shengyu Wang, Zhi‐Yong Liao, Peipei Cao, Marc W. Schmid, Lei Zhang, Jingwen Bi, Stacy B. Endriss, Yujie Zhao, Madalin Parepa, Wenyi Hu, Hikaru Akamine, Jihua Wu, Rui‐Ting Ju, Oliver Bossdorf, Christina L. Richards, Bo Li

**Affiliations:** ^1^ State Key Laboratory of Wetland Conservation and Restoration, National Observations and Research Station for Wetland Ecosystems of the Yangtze Estuary, Ministry of Education Key Laboratory for Biodiversity Science and Ecological Engineering, and Institute of Eco‐Chongming, School of Life Sciences Fudan University Shanghai 200438 China; ^2^ State Key Laboratory of Plant Diversity and Specialty Crops, Xishuangbanna Tropical Botanical Garden Chinese Academy of Sciences Mengla 666303 China; ^3^ MWSchmid GmbH Glarus 8750 Switzerland; ^4^ Department of Natural Resources Cornell University Ithaca NY 14853 USA; ^5^ Department of Environmental Sciences University of North Carolina Wilmington Wilmington NC 28403 USA; ^6^ Department of Entomology Virginia Tech Blacksburg VA 24061 USA; ^7^ Plant Evolutionary Ecology, Institute of Evolution & Ecology University of Tübingen 72076 Tübingen Germany; ^8^ Graduate School of Agriculture University of the Ryukyus 903‐0213 Okinawa Japan; ^9^ Subtropical Field Science Center, Faculty of Agriculture University of the Ryukyus 903‐0213 Okinawa Japan; ^10^ State Key Laboratory of Herbage Improvement and Grassland Agro‐ecosystems, College of Ecology Lanzhou University 730000 Lanzhou China; ^11^ Department of Integrative Biology University of South Florida Tampa FL 33620 USA; ^12^ State Key Laboratory for Vegetation Structure, Functions and Construction, Ministry of Education Key Laboratory for Transboundary Ecosecurity of Southwest China, Institute of Biodiversity, School of Ecology and Environmental Science, and Southwest United Graduate School Yunnan University 650500 Kunming China

**Keywords:** clonality, evolution, genetic accommodation, Japanese knotweed, phenotypic plasticity, plant invasion

## Abstract

- Many widespread invasive plant species express high phenotypic variation across novel environments, providing a unique opportunity to examine ecological and evolutionary dynamics under global change. However, studies often lack information about the origin of introduced populations, limiting our understanding of post‐introduction evolution.
- We assessed the responses of *Reynoutria japonica* from 128 populations spanning latitudinal transects in the native (China and Japan), and introduced (North America and Europe) ranges when grown in two common gardens.
- Plants from introduced populations differed in almost all traits from those from Chinese populations, but were similar to plants from the putative origin in Japan. Compared to Chinese populations, North American, European and Japanese populations expressed lower trait values and plasticity in most traits. However, plants from both introduced and Japanese populations expressed higher clonality and plasticity in clonality than plants from Chinese populations. Further, introduced populations expressed higher plasticity in clonality but lower plasticity in basal diameter compared to Japanese populations.
- Our findings emphasize the potential role of clonality and plasticity in clonality for invasion success. In addition, our study highlights the importance of comparisons to source populations within the native range to identify evolutionary responses of introduced plants to novel environments.

## 摘要

- 许多广泛扩散的入侵植物在新环境中表现出较高的表型变异，这为我们在全球变化背景下研究其生态与进化动态提供了独特的机会。然而，诸多研究往往缺乏引入种群来源的背景信息，因而限制了我们对其引入后进化过程的认识。
- 本研究中，我们沿纬度梯度收集了原产地(中国和日本)和引入地(北美洲和欧洲)的128个虎杖(*Reynoutria japonica*)种群， 比较了其在两个同质园中的生长表现和可塑性。
- 我们发现来自引入地的种群在几乎所有性状表现上均与中国种群存在差异，但与推定的起源地日本种群相似。与中国种群相比，北美、欧洲和日本种群的大多数性状表现出较低的性状值和可塑性。然而，引入地种群和日本种群在克隆性及其可塑性方面表现出高于中国种群的水平。此外，引入地种群的克隆可塑性也高于日本种群，但基径可塑性低于日本种群。
- 我们的研究结果表明，克隆性及其可塑性对外来植物入侵成功可能发挥着关键作用。此外，我们的研究也强调了将引入地种群与原产地源种群进行生物地理学比较的重要性，只有这样才能理解外来植物引种后对新环境的进化响应。

## Introduction

Non‐native species often experience a novel combination of environmental and reproductive barriers in their introduced ranges (Theoharides & Dukes, 2007), providing a unique opportunity to examine rapid ecological and evolutionary dynamics under global change. Understanding the mechanisms that underlie invasion success is also a global imperative, considering that invasive species are ‘main direct drivers of biodiversity loss across the globe’ (Convention on Biological Diversity, 2022). Many hypotheses have proposed that environmental changes in the introduced ranges can influence trait expression, potentially exposing novel trait combinations and driving rapid adaptive evolution (Gioria *et al*., 2023).

Phenotypic plasticity is thought to play an important role in this context either through selection for ‘general‐purpose genotypes’ (Baker, 1965; Bossdorf *et al*., 2005; Richards *et al*., 2006) or as a mechanism to expose cryptic genetic variation that can be refined through genetic accommodation (i.e. the so‐called ‘plasticity‐first’ hypothesis; Levis & Pfennig, 2016). Many studies have shown that introduced populations have greater plasticity than their native conspecifics (Bhattarai *et al*., 2017; Yang *et al*., 2021), but there is a lack of clear support that such plasticity can be translated into fitness benefits (Castillo *et al*., 2021; Boyd *et al*., 2022). As in other studies of plant invasions, an important limitation to support the roles of plasticity in introduced populations is the identification of an appropriate comparison (Van Kleunen *et al*., 2010; Levis & Pfennig, 2016). Comparing introduced populations with their source native populations can improve our understanding of the roles of plasticity in the success of species invasions (Valladares *et al*., 2014; Levis & Pfennig, 2016; Hierro *et al*., 2022).

Studying post‐introduction evolution is further complicated by the fact that some species have large geographical distributions in both native and introduced ranges, requiring careful consideration of the invasion history, which is not well understood for most invasive species (Colautti & Lau, 2015). A growing number of biogeographic studies have shown that similar patterns of abiotic stresses shape parallel clines in plant traits in both native and introduced populations (Maron *et al*., 2004; Van Boheemen *et al*., 2019; Chen *et al*., 2023). Some studies, on the contrary, have found no similarity between clines in native and introduced populations of a single species (Alexander *et al*., 2012; Endriss *et al*., 2018; Liu *et al*., 2020). Therefore, addressing questions about how adaptation of introduced plants differs between continents and during range expansion requires a biogeographic approach (Gioria *et al*., 2023). Common garden experiments have been a useful tool to test for the heritable differences in traits (Moloney *et al*., 2009; Lucas *et al*., 2024). However, the complex history and spread of introduced species dictates careful consideration of the choice of locations for common garden experiments (Moloney *et al*., 2009).

To explore differences in plasticity between native and introduced populations, many studies have manipulated one or a few abiotic factors such as light (Flory *et al*., 2011), nutrients (Luo *et al*., 2019), water availability (Liao *et al*., 2020), herbivory intensity (Sakata *et al*., 2014), or temperature (Molina‐Montenegro & Naya, 2012) in a single common garden. However, the conclusions from such studies depend, at least partly, on where the common garden was located as different sites represent different combinations of diverse biotic and abiotic factors (Maron *et al*., 2007; Woods *et al*., 2012; Yang *et al*., 2021). Further, multiple common gardens are more informative about the differences in responses of plants from native compared to introduced populations (Moloney *et al*., 2009). In addition, asexual clonal growth is envisaged as an important trait that facilitates plant invasions (Atwood & Meyerson, 2011; Wang *et al*., 2017), but evidence of increased clonality has rarely been examined (Bock *et al*., 2018). Consideration of both plasticity and clonality across broad biogeographical ranges may, therefore, help improve our understanding of several hypotheses (Felker‐Quinn *et al*., 2013; Liu *et al*., 2020; Gioria *et al*., 2023), for example, the success of general‐purpose genotypes (Baker, 1965), the importance of phenotypic plasticity (Richards *et al*., 2006; Levis & Pfennig, 2016), and the processes of genetic accommodation (Bock *et al*., 2018).

In previous field observations, we found that plants in introduced populations of *Reynoutria japonica* grew larger and had higher nutritional value compared to conspecific plants in native populations (Irimia *et al*., 2025). Several other studies have reported that introduced populations of *R*. *japonica* consisted of a single genotype in Europe (Hollingsworth & Bailey, 2000; Zhang *et al*., 2016) that also made up most populations in the USA (Richards *et al*., 2012; Gaskin *et al*., 2014; Groeneveld *et al*., 2014). Notably, however, there were at least three introductions in the USA (Del Tredici, 2017). Here, we aimed to clarify the roles of phenotypic plasticity and rapid evolution within the context of the introduction of such a putative ‘general‐purpose genotype’. A ‘general‐purpose genotype’ should be characterized by high phenotypic plasticity, particularly in traits that contribute to invasion success (Baker, 1965; Richards *et al*., 2006). Spread of such genotypes could also be enhanced by clonality (Baker, 1965), and initial plasticity in clonality could expose variation among individuals in the ability to respond with clonal growth (i.e. ‘plasticity first’). In that case, we might expect selection for increased plasticity in clonality (genetic accommodation of plasticity, Bock *et al*., 2018).

To evaluate the contributions of plasticity and clonality to invasion success, we compared growth of *R. japonica* from 55 native‐range populations (Japan and China) and 73 introduced‐range populations (North America and Europe) in two common gardens in China. We tested several alternative hypotheses: (1) plants from introduced ranges exhibit increased growth compared to those from native ranges when grown in common gardens; (2) responses of the introduced populations are more similar to the native Japanese populations (the putative source of the introduced plants) than to the native Chinese populations; (3) the introduced populations have evolved greater phenotypic plasticity than the native populations.

## Materials and Methods

### Study species and collecting sites

*Reynoutria japonica* (Houtt.) Ronse Decraene (Japanese knotweed, Polygonaceae) is native to eastern Asia (Bailey & Conolly, 2000). This species was introduced into Europe in the mid‐19^th^ century (Beerling *et al*., 1994; Bailey & Conolly, 2000) and into North America before 1873 (Barney, 2006). Because of the highly negative effects of *R. japonica*, it has been listed as one of the 100 world's worst invasive alien species by the IUCN (Lowe *et al*., 2000). Introduced populations of *R. japonica* have almost no genetic diversity, and are thought to originate from a single female individual (Hollingsworth & Bailey, 2000; Richards *et al*., 2012; Gaskin *et al*., 2014; Groeneveld *et al*., 2014; Jugieau *et al*., 2024; but see VanWallendael *et al*., 2021). We recently confirmed that all plants we sampled in Europe and most populations we sampled in the USA shared the same chloroplast haplotype (Zhang *et al*., 2024).

**Fig. 1:**
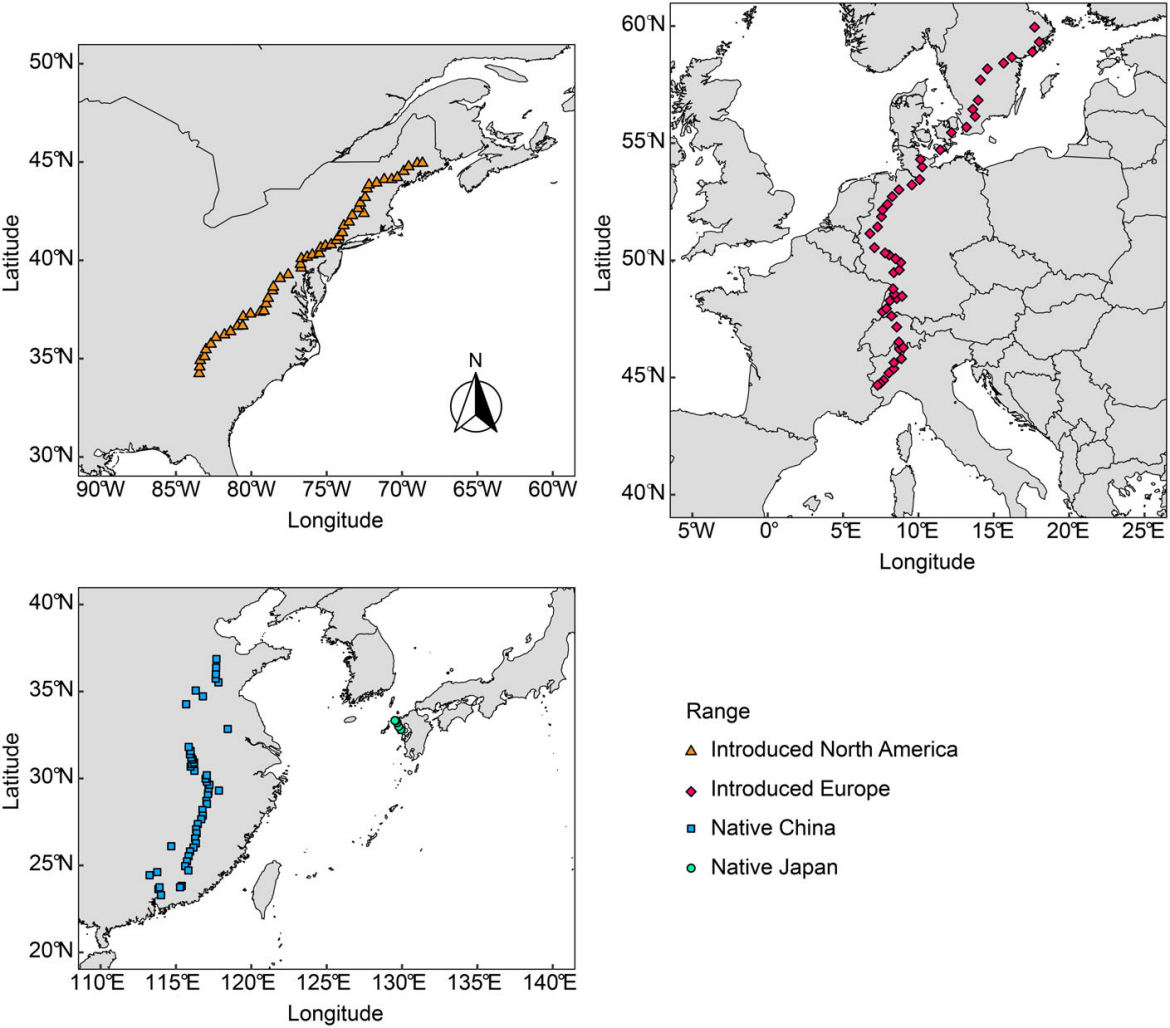
Locations of *Reynoutria japonica* populations sampled in the field. We sampled 50 populations along latitudinal transects in the introduced North American range, introduced European range, and native Chinese range. We sampled six populations in native Japanese range from the area that is considered to be the source of the introduced populations. See Supporting Information Table S1 for details about the 128 populations.

From 2019 to 2020, we conducted a cross‐latitudinal survey of 50 populations of *R. japonica* approximately evenly spaced along a 2000 km transect in each of the native range of China and the introduced ranges of North America and Europe (Fig. 1; Irimia *et al*., 2025). At each site, we collected rhizome pieces from five individuals at 2, 8, 14, 20, and 26 m along a 30 m transect. For populations smaller than 30 m in length, we reduced the distance between the individuals but the rhizomes we sampled were always separated by at least 1 m (see more details in Irimia *et al*., 2025). In addition, we collected rhizomes from six populations in Nagasaki, Japan, separated by *c*. 20 km because this area was known as the potential source of North American and European populations (Beerling *et al*., 1994; Bailey & Conolly, 2000). We collected rhizome pieces from 5 to 8 individuals from each Japanese population between 28 April and 4 May 2021. Our chloroplast sequencing data showed that the Japanese populations we collected share the same chloroplast haplotype as the introduced North American and European populations (Zhang *et al*., 2024). By contrast, we found 26 haplotypes in the Chinese populations that we sampled, none of which overlapped with the one in the introduced and Japanese populations (Zhang *et al*., 2024). We, therefore, assumed the Japanese populations were the source within the native range (Zhang *et al*., 2024).

In 2021, we imported and grew the rhizome fragments from Japan, Europe, and North America under quarantine conditions in a glasshouse for one growing season at Xishuangbanna Tropical Botanical Garden, Chinese Academy of Sciences (Mengla, Yunnan, China). Meanwhile, we also grew the Chinese rhizomes under the same glasshouse conditions. Due to import limitations, we obtained only one individual from each North American population. After collection, we excluded some individuals from the introduced ranges that we discovered were the hybrid species *Reynoutria × bohemica* based on genome size and ploidy level data (Irimia *et al*., 2025). In addition, several *R. japonica* individuals failed to sprout. Therefore, from the original 156 populations, we ended up with a total of 524 *R. japonica* individuals from 128 populations: 240 from 50 Chinese, 34 from 5 Japanese, 27 from 27 North American, and 223 from 46 European populations (Supporting Information Table S1).

In February 2022, we cut two pieces of rhizomes from each individual and stored them at 4°C for at least 4 wk to facilitate sprouting success. One day before we setup the common gardens, we removed the fine roots of each rhizome, and trimmed them to similar size with at least one bud to ensure sprouting and minimize maternal effects. We weighed each rhizome piece to include in the analysis as a covariate.

### Experimental setup

We established two common gardens: one at Xishuangbanna Tropical Botanical Garden, Chinese Academy of Sciences, Yunnan (XTBG; 101°16′E, 21°54′N) and the other at Fudan University, Shanghai (SH; 121°30′E, 31°20′N). The XTBG garden is close to the southern limit of the native range, and the SH garden is roughly at the distributional center of the native range. XTBG garden has a tropical monsoon climate characterized as warmer and moister during the growing season compared to the moderate temperate‐zone climate of Shanghai garden. To visualize these differences in the context of our collections, we obtained recent climate data (the 1970–2000 averages) for the 128 populations and the two common garden locations from WorldClim 2 (Fick & Hijmans, 2017), at a spatial resolution of 30 arc seconds. We used the 19 bioclimatic variables (accessible at: https://www.worldclim.org/data/bioclim.html), which cover different aspects of temperature and precipitation and their temporal variability. In addition, we recently reported that the herbivory load was higher in XTBG garden compared to that of SH garden (Cao *et al*., 2025). Hence, the differences between the two gardens reflected a combination of abiotic and biotic conditions.

We used identical plant cultivation methods in each garden (also see Cao *et al*., 2025), including potting substrates and fertilization. On 11 March 2022, we treated all the rhizomes with fungicide and transplanted them separately into outdoor 20 l plastic pots with standard soil (Pindstrup substrate 5–20 mm; Pindstrup Mosebrug A/S, Pindstrup, Denmark). We used large pots to minimize the potential growth limitation related to pot size (Megersa *et al*., 2018). We randomly arranged all pots into five blocks within each garden, with one individual from each population in each block for European and Chinese populations. For the Japanese and the North American populations, we randomly assigned an even number of pots into each of the blocks (5–7 in each block). The distance between neighbor pots was at least 90 cm to avoid aboveground interference. We added 10 g Osmocote fertilizer (Osmocote plus 801, N : P : K 16 : 8 : 12; Everris International B.V., Heerlen, the Netherlands) onto the soil surface in each pot at the beginning and mid‐point of the experiment. Throughout the experiment, we watered the plants when needed. We put a saucer under each pot to avoid water and nutrient loss, and removed mature seeds during our daily observations to prevent potential spread in the field. Due to limited rhizome availability and growth success for some individuals, we eventually measured traits of 464 individuals of *R. japonica* at the XTBG garden (233 individuals from 50 Chinese populations, 25 individuals from 5 Japanese populations, 190 individuals from 46 European populations, and 16 individuals from 16 North American populations) and 519 individuals at the SH garden (240 from 50 Chinese populations, 34 from 5 Japanese populations, 218 from 46 European populations, and 27 from 27 North American populations).

### Trait measurements

At the peak of the growing season in July 2022, we counted the number of ramets (our estimate of clonality) that were > 5 cm tall in each pot. We also scanned the top 5 fully expanded leaves of the tallest ramet (Epson Perfection V550, Epson, Suwa, Japan), and calculated the total leaf area of the five leaves after subtracting any area missing due to herbivory damage (with Adobe Photoshop using ImageJ; Schneider *et al*., 2012) as leaf size. In October 2022, we measured the basal diameter and height of the tallest ramet in each pot, as most of the plants had turned yellow and had reached the end of the growing season. We then harvested all aboveground parts and dried all plant materials at 75°C for at least 72 h before determination of dry mass. We did not count the number of ramets again at the final harvest as we found almost no new ramets sprouted after the peak of the growing season. All data are available online (Wang *et al*., 2024).

### Statistical analyses

We performed all analyses in R 4.3.2 (R Core Team, 2023). To visualize the differences in climate across the source populations and common gardens, we conducted a standardized and centered principal component analysis (PCA) using the prcomp function and generated a PCA biplot of all populations in the climatic space to evaluate climatic variable contributions to the principal components. In addition, to visualize the overall differences in plant traits among ranges, we used two separate Horn's parallel analyses to perform PCA on all measured traits in each of the two common gardens (Revelle, 2024). For each PCA, we used the mean trait value within a garden for each population (i.e. one data point per population).

To evaluate differences in trait means and plasticities among ranges, we used linear models and all data from the four ranges in the two gardens (i.e. 983 plants). We fitted factors sequentially (type I sum of squares) following general design principles (Schmid *et al*., 2017) as indicated in Table 1. Significance tests were based on *F* tests using appropriate error terms and denominator degrees of freedom (Schmid *et al*., 2017). The fixed terms of the models were initial fresh mass of rhizome, garden, range, and their interactions. The random terms were population and the interaction between garden and population. The factor ‘range’ was further split into three contrasts with one degree of freedom each (Schmid *et al*., 2017): the first compared CN to the other three ranges, the second compared JA to EU and US, and the third compared EU to US. Given that population was treated as a random term, the factor range and its contrasts were tested against the factor population. Likewise, the interaction of the factor range and its contrasts with the factor garden were tested against the interaction between population and garden. In this model, the main effect of range and its contrast compared trait means, and the interactions compared trait plasticities. To compare all pairwise‐differences in trait means within each garden, we used the same model structure to run linear mixed models implemented in the lme4 package (Bates *et al*., 2015), along with Tukey's Honestly Significant Difference tests (Tukey's HSD) implemented in the emmeans package (Lenth, 2022).

**Table 1:** The effects of rhizome mass (covariate), range, garden and their interactions on plant traits of *Reynoutria japonica* shown as the percent sum of explained by each term.

Term	df	Height	Diameter	Leaf size	Leaf mass	Stem mass	Above. mass	No. ramets
Rhizome mass	1	3.26***	3.32***	5.12***	0.33***	1.09***	0.73***	2.12***
Garden	1	24.53***	50.89***	5.88***	78.37***	63.26***	80.78***	7.28***
Range, contrasts	3	38.62***	15.45***	25.94***	0.29 ns	10.05***	0.43 ns	47.9***
CN vs JA/EU/US	1	38.55***	14.94***	25.64***	0.28^#^	10.01***	0.42*	47.72***
JA vs EU/US	1	0.01 ns	0.43**	0.01 ns	0.01 ns	0.03 ns	0 ns	0.12^#^
EU vs US	1	0.06 ns	0.07 ns	0.3 ns	0.01 ns	0 ns	0 ns	0.06 ns
Population	123–124	9.32***	7.86***	20.95***	8.76***	10.75***	8.48***	5.07 ns
Garden: Range, contrasts	3	0.23 ns	3.78***	2.73***	0.05 ns	3.63***	0.27**	7.62***
Garden: CN vs JA/EU/US	1	0.15^#^	3.48***	2.4***	0.01 ns	3.63***	0.24**	6.95***
Garden:JA vs EU/US	1	0.09 nfs	0.29**	0.02 ns	0.03 ns	0 ns	0.02 ns	0.67***
Garden: EU vs US	1	0 ns	0.01 ns	0.32^#^	0.01 ns	0 ns	0 ns	0 ns
Garden: Population	112–113	4.85***	2.93^#^	10.02***	2.85***	3.1***	2.44***	2.05 ns
Residuals	706–737	19.18 ns	15.78 ns	29.36 ns	9.35 ns	8.13 ns	6.87 ns	27.96 ns

Statistical significance is indicated as: ns, *P* ≥ 0.1; #, *P* < 0.1; *, *P* < 0.05; **, *P* < 0.01; ***, *P* < 0.001. Above. mass, aboveground biomass; df, degrees of freedom. *P*‐values were calculated by treating population as a random factor. Thus, the factor range and its contrasts were tested against population, and the interactions of range and its contrasts with garden against garden:population. Garden was tested against the residuals. Even though population was treated as a random factor, we provided the variation explained by it and the results of a test against residuals.

## Results

We found that on average, the trait means and plasticity of introduced populations differed from those of native Chinese populations but were very similar to those of Japanese populations. An important exception was the increased plasticity in clonality in introduced populations compared to Japanese populations.

### Plant trait divergence among ranges

We found large overlap in climates within the two native and within the two introduced ranges in the PCA of climate data, but a clear separation between native and introduced ranges by PC1. PC1 was largely associated with mean temperatures, and precipitation in the wettest periods (Fig. S1). The XTBG and SH gardens represented variation in both PC1 and PC2, but did not include the full range of variation in PC1 (i.e. did not include the variation in PC1 found in the introduced ranges). The gardens were separated by PC2, which was associated with temperature seasonality and temperature annual range.

**Fig. 2:**
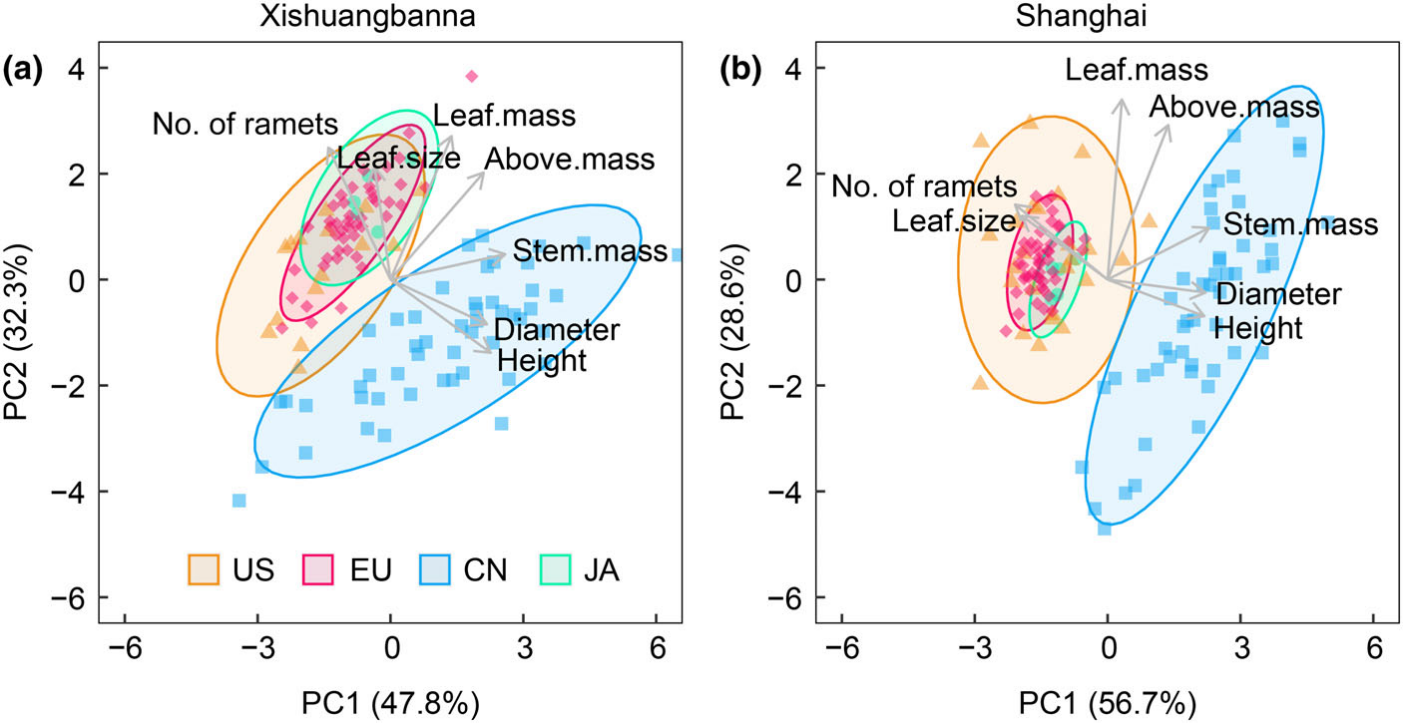
Biplots of principal components analysis of plant traits of *Reynoutria japonica* measured in the Xishuangbanna (a) and Shanghai (b) common gardens. Plant trait loading vectors are plotted as arrows. Above.mass, aboveground biomass; CN, native China; EU, introduced Europe; JA, native Japan; Leaf.mass, leaf biomass; Stem.mass, stem biomass; US, introduced North America.

The first two components of the PCA of plant traits explained 80.1% and 85.3% of the trait variation across all four ranges for plants grown in the XTBG (Fig. 2a) and SH (Fig. 2b) common gardens, respectively. In contrast to the climate PCA, the plant trait analysis clearly showed that plants from Chinese populations separated from those in the other three ranges in both gardens. Plants from Japan, North America and Europe largely overlapped in both gardens in multivariate trait space.

**Fig. 3:**
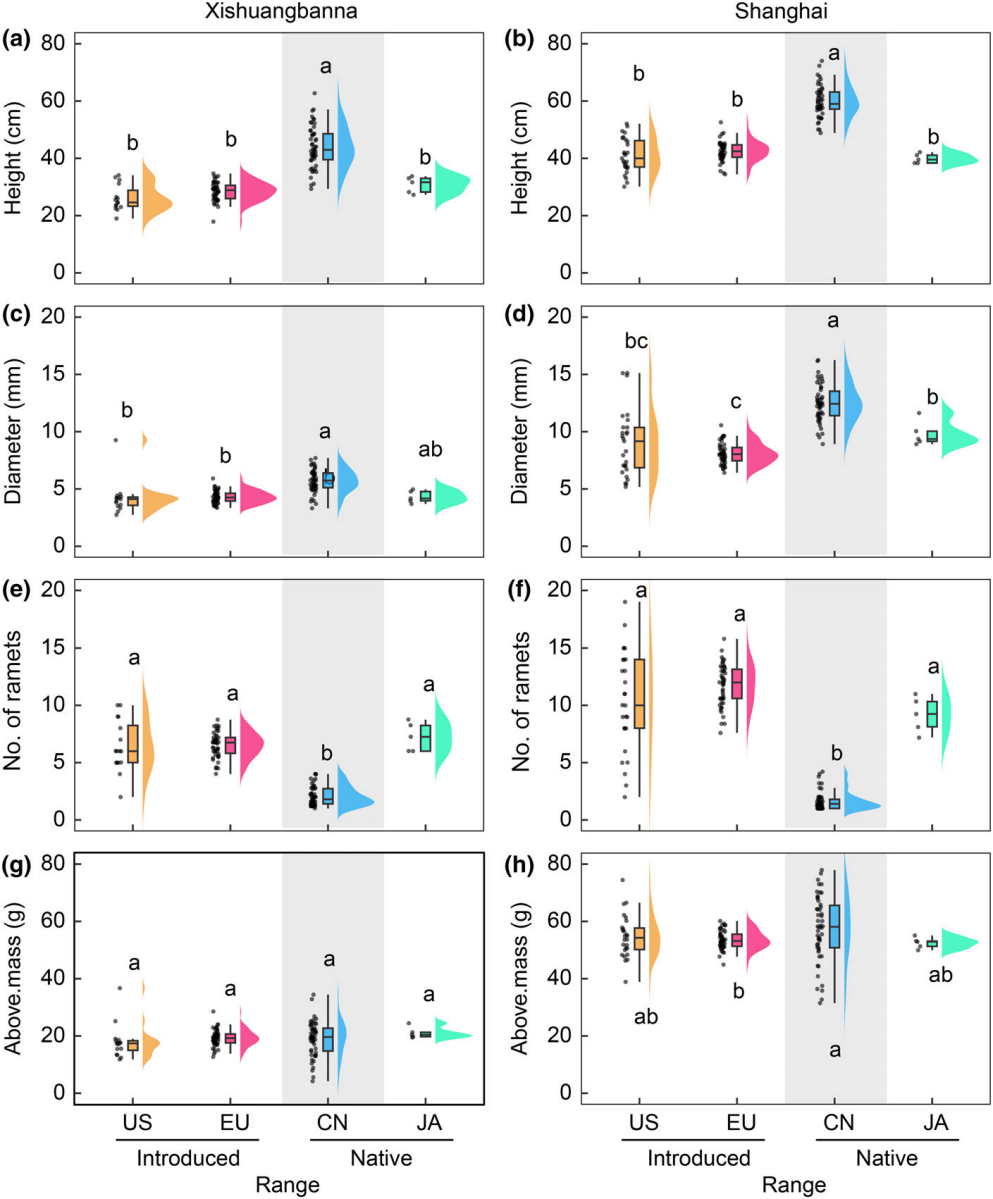
Variation in plant traits of *Reynoutria japonica* from introduced North American (US) and European (EU) and native Chinese (CN) and Japanese (JA) ranges in Xishuangbanna (a, c, e, g) and Shanghai (b, d, f, h) common gardens. Graphs include population mean values (points), boxplots (centerline, median; box limits, upper and lower quartiles; whiskers, 1.5× interquartile range; points, outliers), and violin plot based on Kernel density function. Different letters represent significant differences (*P* < 0.05 with Tukey's Honestly Significant Difference tests implemented in the emmeans package) in plant trait values among ranges. Above.mass, aboveground biomass. We added grey shading in each panel to emphasize the difference between CN and the other three ranges.

The separation of Chinese populations from those from Japan, North America and Europe was recapitulated in analyses of trait responses. Plants from the native Japanese populations were not significantly different from those from introduced North American and European populations in mean for most traits (except basal diameter in Shanghai; Fig. 3d; Table S2). Plants from Japanese and introduced populations were shorter and thinner but had more ramets than those from Chinese populations (Fig. 3a–f). We found no significant differences in aboveground biomass among ranges, except for slightly higher biomass in Chinese plants compared to European plants in the SH garden (Fig. 3g,h).

### Divergence in phenotypic plasticity

**Fig. 4:**
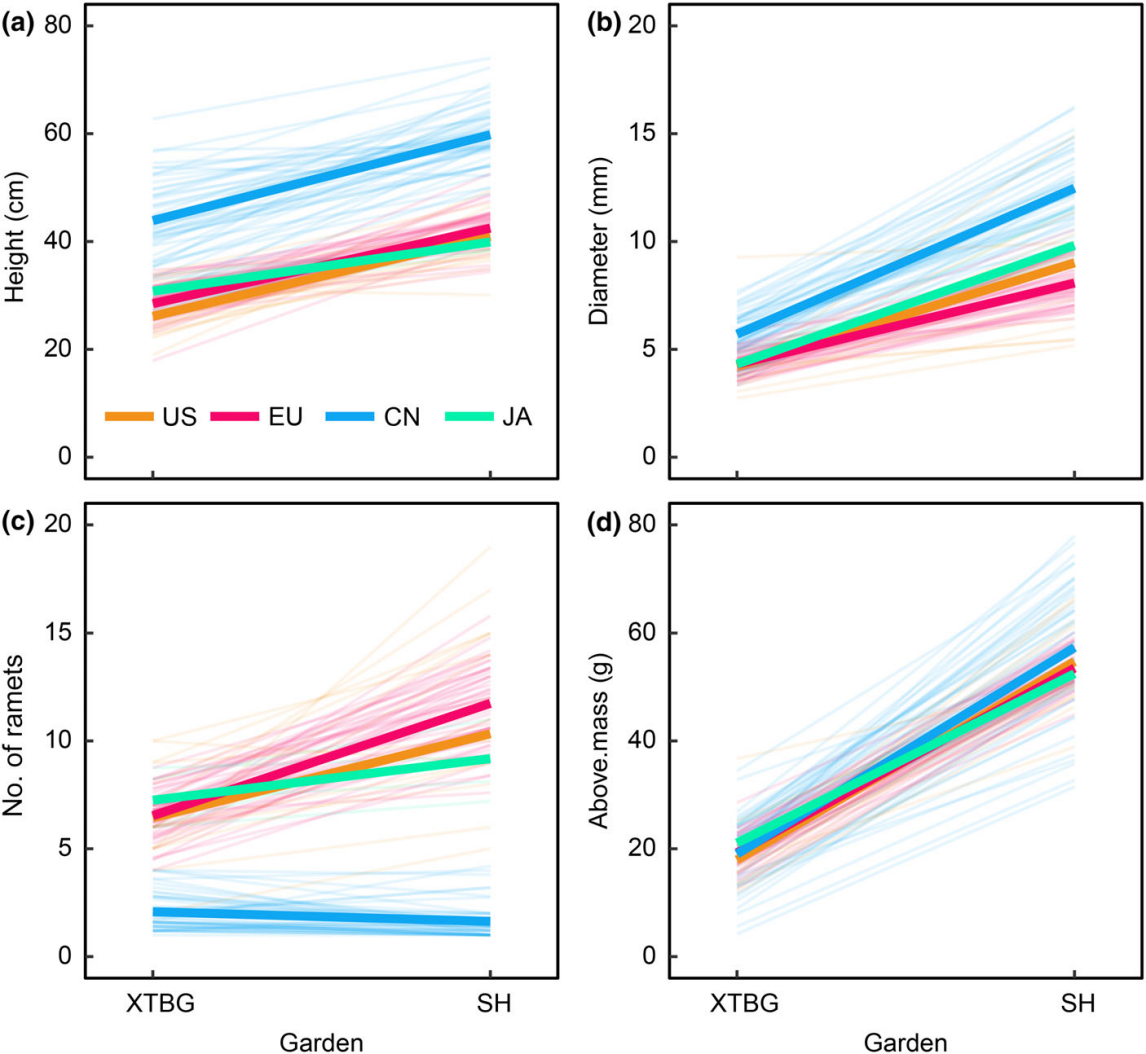
Reaction norms of plant height (a), basal diameter (b), number of ramets (c) and aboveground biomass (d) for populations of *Reynoutria japonica* from introduced North American (US) and European (EU) and native Chinese (CN) and Japanese (JA) ranges grown in Xishuangbanna (XTBG) and Shanghai (SH) common gardens. Mean response for each population is indicated by thin lines. Mean response for each range is indicated by thick lines. Above.mass, aboveground biomass. See more details of statistical significance in Table 1.

We found significant range by garden effects for almost all traits (Tables 1, S2). Plasticity in plant height, basal diameter and aboveground biomass was lower for the Japanese and introduced populations compared to Chinese populations (Fig. 4a,b,d). Plants from Japanese and introduced populations had higher plasticity in number of ramets compared to the native Chinese populations, and plants from introduced populations had higher plasticity than those from Japan (Fig. 4c).

## Discussion

Introduced species around the globe provide unique opportunities to examine mechanisms that allow for rapid responses to novel and changing environmental conditions (Lee, 2002; Estoup *et al*., 2016). Many different mechanisms – including introduction of ‘pre‐adapted’ genotypes, and the evolution of plant traits in response to novel environments – have been examined to explain the biogeographic differences of invasive species (Callaway *et al*., 2022; Hierro *et al*., 2022; Gioria *et al*., 2023). In previous work, we found that plants in the field in introduced *R. japonica* populations were larger with higher nutritional value compared to conspecifics in native populations (Irimia *et al*., 2025). Our results here showed that when grown in common gardens, the same *R. japonica* plants we sampled in the introduced ranges had not evolved larger sizes, which did not support our first hypothesis. In fact, we found that plants from all four ranges had nearly equivalent aboveground biomass in both gardens. However, we found support for our second hypothesis because introduced plants were not differentiated from those from the putative native source Japanese populations in most traits. Plants from China were taller and had fewer ramets than plants from Japan, North America and Europe, reflecting broad differences in the native range of this species. We also found support for our third hypothesis related to evolution of plasticity because we were able to evaluate plants from the native source populations of Japan separately from the native populations in China. Both introduced North American and European populations were more plastic in number of ramets than putative source populations in response to growing conditions of our common garden sites, indicating a post‐introduction evolution of higher clonality in the introduced ranges.

Our results suggested that the introduction of a highly clonal genotype (general‐purpose genotype) from Japan might have contributed to the invasion success of *R. japonica*. In our experiment, we found that introduced plants consistently produced many more ramets than those from Chinese populations but were similar to plants from Japanese populations. This finding is in line with reports that the strong vegetative reproductive ability of introduced *R. japonica* underlies its expansion into more habitats in its introduced ranges (Beerling *et al*., 1994; De Waal, 2001; Cao *et al*., 2024). In general, native Chinese populations were taller and produced fewer ramets than introduced and native Japanese genotypes, which might reflect an evolutionary history that favors height growth over clonal reproduction in the Chinese populations (Van Kleunen *et al*., 2001; Bossdorf *et al*., 2004).

Phenotypic plasticity is thought to be useful to founding populations by extending niche breadth (Baker, 1965; Bossdorf *et al*., 2005; Richards *et al*., 2006), and could be important for adaptation to global change and increased unpredictability of climatic anomalies (Nicotra *et al*., 2010; Vázquez *et al*., 2017). However, phenotypic plasticity is the property of specific traits and its importance can vary across traits and environmental contexts (Richards *et al*., 2006). We found large phenotypic changes in response to growing conditions of our common garden sites, but Chinese plants were more plastic in plant height, basal diameter and aboveground biomass than introduced or Japanese plants. We also found higher plasticity in basal diameter in plants from Japan than those from the introduced ranges in North America and Europe. The results suggest that plasticity in these traits may not provide an advantage in the introduced ranges.

Nevertheless, our experiment did support the plasticity‐first hypothesis and the possibility of genetic accommodation of increased clonality. We showed that the introduced plants exhibited traits that were not different from those of the presumed source of the introduction (Japanese populations) in each of the two common gardens. The introduced plants were also not different from Japanese plants in plasticity of most traits. However, our results supported increased plasticity in number of ramets from both introduced ranges compared to the native Japanese populations. In sum, our findings provided evidence that an evolutionary shift to increased plasticity in clonality, or genetic accommodation of clonality, might have facilitated the invasion success of *R. japonica* (Levis & Pfennig, 2016; Bock *et al*., 2018).

Considering the size and ploidy levels of the knotweed genome, plasticity to novel conditions in the introduced ranges may have exposed cryptic genetic variation that was not expressed in the native range (i.e. the so‐called ‘plasticity‐first’ hypothesis; Levis & Pfennig, 2016; Mounger *et al*., 2021). Then, selection could have acted on this variation and increased standing levels of plasticity in the introduced populations. Another study found support for increased ability to respond specifically to well‐watered conditions by producing more clonal propagules in *Helianthus tuberosus* (Bock *et al*., 2018). They found evidence for hybrid vigor in the introduced populations and two specific quantitative trait loci (QTL) that were associated with the increased ability to respond to water content in the introduced habitat. On the other hand, several studies have reported that somatic mutation may allow asexual species to maintain abundant genetic variation and adapt to changing environmental conditions (reviewed in Schoen & Schultz, 2019; see also discussions in Chen *et al*., 2020; Robertson *et al*., 2020).

The increased plasticity in *R. japonica* could have resulted from novel outcrossing events or additional introductions. We do not expect that such events contributed to our results considering our recent findings that all *R. japonica* plants from introduced populations that we used in our study had the same chloroplast haplotype as those from the Japanese populations we sampled (Zhang *et al*., 2024). VanWallendael *et al*. (2021) reported genetic diversity in other North American *R. japonica* populations, but they also discussed the limitations of their findings considering the characteristics of the *R. japonica* genome. In particular, within‐individual heterozygosity is difficult to differentiate from among‐individual single nucleotide polymorphisms (SNPs) in a large octoploid genome. Identifying true polymorphisms from within‐individual polymorphisms or sequencing error requires much greater sequencing depth than most studies allow for. In addition, VanWallendael *et al*. (2021) reported lower SNP diversity in *R. japonica* than *R. × bohemica* or *R. sachalinensis* and all of these taxa are expected to have very low diversity levels. Further work and more genomic resources for *R. japonica* are required to link specific genomic changes in this species that allowed for the increase in plasticity of clonality.

We acknowledge that these findings are preliminary given some of the limitations in our study. For example, plants from the four ranges accumulated similar aboveground biomass, implying two distinct trait strategies to achieve the same level of fitness in the common garden environments during this growing season. Many individuals may not flower at all in the field, but persist and spread from year to year so biomass is an important indicator of fitness (Yuan *et al*., 2024). However, our study did not directly relate these traits to competitive ability (e.g. competitive suppression or competitive tolerance). In addition, the common garden sites in our experimental setup could represent extreme conditions for some populations, especially in the XTBG experimental sites in southern China. Ideally, we would compare common gardens of the full collection of native and introduced *R. japonica* in North America and Europe to evaluate the conditions in the introduced range, but, due to ethical constraints, it is not feasible to establish such gardens. We would also ideally assess replicates of more than one individual for each of the USA populations which may partly explain the larger variation in plant traits in the USA. The fact that we were able to identify differences in plasticity despite this delimited design suggests that the results should be conservative. Finally, although we have some evidence of history and phylogenetic relationships of the plants we used, we collected a limited number of Japanese populations and could not explicitly verify the source population in Japan. Future work should sample Japanese populations more extensively. Despite these limitations, we elicited plant trait differences among four ranges across the two common gardens, which reflects biological differences among populations of this globally invasive species. Further work is needed to confirm whether the differences we identified have contributed to the competitive advantage of the introduced populations (Alexander & Levine, 2019).

### Conclusions

Although comparisons of plant traits between native and introduced populations have been widely used to examine the rapid evolution of invasive plants, the differences in growth and reproduction traits between introduced plants and those from source and non‐source native populations have seldom been tested at the same time. Our study allowed us to examine evolutionary changes in trait means and plasticities in two distinct common garden environments. A novel finding is that the trait expressions in growth and reproduction of introduced plants were very similar to those from the putative source populations, but not other native populations. Introduced populations may not express higher growth performance than all native populations, which would challenge the idea that evolution of increased competitive ability is universal in invasive plants. In the case of *R. japonica*, plants from the native source and introduced populations also exhibit higher plasticity in clonality than native non‐ source populations, suggesting that the introduction of general‐purpose genotypes may have enhanced the potential for establishment and invasion into wider ranges. Moreover, our results show that the evolutionary shift of higher plasticity in clonality, but not other traits, may further facilitate the large‐scale colonization of *R. japonica* in introduced ranges. Our ability to identify the potential role of clonality and plasticity of these plants highlights the importance of combining the invasion history of widely distributed native and introduced populations when evaluating evolutionary responses (Hierro *et al*., 2005; Colautti & Lau, 2015).

## Competing interests

None declared.

## Author contributions

SW provided the conceptualization, data curation, formal analysis, investigation, methodology, project administration, resources, validation, visualization, original draft preparation, review and editing. Z‐YL provided the conceptualization, data curation, formal analysis, investigation, methodology, project administration, resources, validation, visualization, review and editing. PC provided the investigation, project administration, resources, review and editing. MWS provided the formal analysis, methodology, review and editing. LZ provided the investigation, resources, review and editing. JB provided the investigation, resources, review and editing. SBE was involved in review and editing. YZ was involved in investigation, resources, review and editing. MP was involved in visualization, review and editing. WH and HA provided the resources, review and editing. JW and R‐TJ were involved in conceptualization, review and editing. OB provided the conceptualization, funding acquisition, project administration, review and editing. CLR provided the conceptualization, formal analysis, funding acquisition, project administration, review and editing and supervision. BL provided the conceptualization, funding acquisition, project administration, review and editing and supervision.

## Disclaimer

The New Phytologist Foundation remains neutral with regard to jurisdictional claims in maps and in any institutional affiliations.

## Supporting information


**Fig. S1** Biplot of principal component analysis using environmental variables at collection sites of *Reynoutria japonica* populations used in our study and at the two common garden sites.
**Table S1** Geographic origins of the populations.
**Table S2** The effects of range, garden and their interactions on plant traits.
Please note: Wiley is not responsible for the content or functionality of any Supporting Information supplied by the authors. Any queries (other than missing material) should be directed to the *New Phytologist* Central Office.

## Data Availability

The data that support the findings of this study are openly available in Figshare at doi: 10.6084/m9.figshare.27216120.
