# Supplementary material for: General‐purpose genotypes and evolution of higher plasticity in clonality underlie knotweed invasion
Source: New Phytol. 2025 Feb 19;246(2):758–68. doi: 10.1111/nph.20452 (PMC11923409; doi:10.1111/nph.20452)
Supplement: Supplementary file 1 — Fig. S1 Biplot of principal component analysis using environmental variables at collection sites of Reynoutria japonica populations used in our study and at the two common garden sites. Table S1 Geographic origins of the populations. Table S2 The effects of range, garden and their interactions on plant traits. Please note: Wiley is not responsible for the content or functionality of any Supporting Information supplied by the authors. Any queries (other than missing material) should be directed to the New Phytologist Central Office. [file NPH-246-758-s001.pdf]

New Phytologist

New Phytologist Supporting Information

Article title: General-purpose genotypes and evolution of higher plasticity in clonality underlie knotweed invasion
Authors: Shengyu Wang, Zhi-Yong Liao, Peipei Cao, Marc W. Schmid, Lei Zhang, Jingwen Bi, Stacy B. Endriss, Yujie Zhao, Madalin Parepa, Wenyi Hu, Hikaru Akamine, Jihua Wu, Rui-Ting Ju, Oliver Bossdorf, Christina L. Richards, Bo Li
Article acceptance date: 18 January 2025

The following Supporting Information is available for this article:

Table S1 Geographic origins of the 128 studied populations of *Reynoutria japonica* and their uses in the Xishuangbanna Tropical Botanical Garden (XTBG) and Shanghai (SH) common gardens.

Range	Population ID	Garden	Latitude	Longitude
US	US01	XTBG/SH	34.24	-83.46
US	US04	XTBG/SH	35.1	-83.1
US	US06	XTBG/SH	35.74	-82.68
US	US08	XTBG/SH	36.21	-81.78
US	US09	XTBG/SH	36.38	-81.38
US	US10	XTBG/SH	36.65	-80.92
US	US11	SH	36.67	-80.57
US	US14	SH	37.37	-79.41
US	US15	SH	37.43	-79.16
US	US18	XTBG/SH	38.46	-78.59
US	US19	SH	38.65	-78.54
US	US20	XTBG/SH	39.08	-78.09
US	US22	XTBG/SH	39.61	-76.68
US	US31	XTBG/SH	41.01	-74.35
US	US33	XTBG/SH	41.41	-73.96
US	US34	SH	41.77	-73.86
US	US38	XTBG/SH	42.64	-72.91
US	US39	XTBG/SH	42.92	-72.77

US	US40	XTBG/SH	43.2	-72.45
US	US42	SH	43.84	-72.19
US	US43	XTBG/SH	43.94	-71.68
US	US44	XTBG/SH	44.12	-71.18
US	US45	SH	44.11	-70.69
US	US46	SH	44.21	-70.31
US	US47	SH	44.53	-69.89
US	US49	SH	44.94	-68.99
US	US50	SH	44.95	-68.64
EU	EU01	XTBG/SH	44.88	7.69
EU	EU02	XTBG/SH	44.75	7.48
EU	EU03	XTBG/SH	44.67	7.29
EU	EU04	XTBG/SH	45.19	8.04
EU	EU06	XTBG/SH	45.64	8.37
EU	EU07	XTBG/SH	45.8	8.87
EU	EU09	XTBG/SH	46.27	9
EU	EU10	XTBG/SH	46.51	8.7
EU	EU11	XTBG/SH	47.16	8.56
EU	EU12	XTBG/SH	47.62	8.22
EU	EU13	XTBG/SH	47.81	7.61
EU	EU14	XTBG/SH	47.94	7.88
EU	EU15	XTBG/SH	48.28	8.11
EU	EU16	XTBG/SH	48.37	8.56
EU	EU17	XTBG/SH	48.47	8.92
EU	EU18	XTBG/SH	48.56	8.39
EU	EU19	XTBG/SH	48.79	8.32
EU	EU21	XTBG/SH	49.59	8.73
EU	EU22	XTBG/SH	49.9	8.83
EU	EU23	XTBG/SH	50.07	8.48
EU	EU25	XTBG/SH	50.31	7.79
EU	EU26	XTBG/SH	50.54	7.08
EU	EU27	XTBG/SH	51.14	6.78

EU	EU28	XTBG/SH	51.43	7.29
EU	EU29	XTBG/SH	51.87	7.55
EU	EU30	XTBG/SH	52.15	7.62
EU	EU31	XTBG/SH	52.39	7.94
EU	EU32	XTBG/SH	52.72	8.26
EU	EU33	XTBG/SH	53.01	8.7
EU	EU34	XTBG/SH	53.22	9.57
EU	EU35	XTBG/SH	53.45	10.08
EU	EU36	XTBG/SH	53.99	10.25
EU	EU37	XTBG/SH	54.31	10.13
EU	EU38	XTBG/SH	54.71	11.45
EU	EU39	XTBG/SH	55.46	12.19
EU	EU40	XTBG/SH	55.68	13.19
EU	EU41	XTBG/SH	56.14	13.76
EU	EU42	XTBG/SH	56.45	13.6
EU	EU43	XTBG/SH	56.83	13.96
EU	EU44	XTBG/SH	57.7	14.11
EU	EU45	XTBG/SH	58.17	14.58
EU	EU46	XTBG/SH	58.41	15.65
EU	EU47	XTBG/SH	58.67	16.2
EU	EU48	XTBG/SH	58.89	17.56
EU	EU49	XTBG/SH	59.32	18.02
EU	EU50	XTBG/SH	59.95	17.71
CN	CN01	XTBG/SH	29.31	117.85
CN	CN02	XTBG/SH	29.21	117.15
CN	CN03	XTBG/SH	28.71	117.02
CN	CN04	XTBG/SH	28.54	117.05
CN	CN05	XTBG/SH	28.19	116.76
CN	CN06	XTBG/SH	27.87	116.78
CN	CN07	XTBG/SH	27.66	116.68
CN	CN08	XTBG/SH	27.38	116.45
CN	CN09	XTBG/SH	27.08	116.34

CN	CN10	XTBG/SH	26.85	116.37
CN	CN11	XTBG/SH	26.55	116.27
CN	CN12	XTBG/SH	26.27	116.32
CN	CN13	XTBG/SH	26.03	116.16
CN	CN14	XTBG/SH	25.8	115.94
CN	CN15	XTBG/SH	25.53	115.84
CN	CN16	XTBG/SH	25.24	115.73
CN	CN17	XTBG/SH	24.96	115.61
CN	CN18	XTBG/SH	24.71	115.82
CN	CN19	XTBG/SH	23.82	115.38
CN	CN20	XTBG/SH	23.75	115.27
CN	CN21	XTBG/SH	23.64	113.84
CN	CN22	XTBG/SH	23.29	114.01
CN	CN23	XTBG/SH	23.74	113.91
CN	CN24	XTBG/SH	24.44	113.25
CN	CN25	XTBG/SH	24.62	113.76
CN	CN26	XTBG/SH	26.1	114.69
CN	CN27	XTBG/SH	29.1	117.12
CN	CN28	XTBG/SH	29.43	117.16
CN	CN29	XTBG/SH	29.64	117.22
CN	CN30	XTBG/SH	29.81	117.05
CN	CN31	XTBG/SH	29.98	116.97
CN	CN32	XTBG/SH	30.18	117.04
CN	CN33	XTBG/SH	30.44	116.23
CN	CN34	XTBG/SH	30.69	115.98
CN	CN35	XTBG/SH	30.82	116.05
CN	CN36	XTBG/SH	30.92	116.21
CN	CN37	XTBG/SH	31.09	116.1
CN	CN38	XTBG/SH	31.21	116.02
CN	CN39	XTBG/SH	31.4	115.93
CN	CN40	XTBG/SH	31.58	115.97
CN	CN41	XTBG/SH	31.81	115.85

CN	CN42	XTBG/SH	34.27	115.68
CN	CN43	XTBG/SH	34.72	116.79
CN	CN44	XTBG/SH	35.06	116.31
CN	CN45	XTBG/SH	35.52	117.82
CN	CN46	XTBG/SH	35.74	117.64
CN	CN47	XTBG/SH	36	117.65
CN	CN48	XTBG/SH	36.38	117.66
CN	CN49	XTBG/SH	36.87	117.68
CN	CN50	XTBG/SH	32.85	118.44
JA	JA02	XTBG/SH	32.81	129.92
JA	JA03	XTBG/SH	32.99	129.77
JA	JA04	XTBG/SH	33.21	129.68
JA	JA05	XTBG/SH	33.32	129.6
JA	JA06	XTBG/SH	33.33	129.53

New Phytologist

Table S2 The effects of range, garden and their interactions on plant traits of *R. japonica*. Df, Degrees of freedom; SS, Sum of squares. P-values were calculated by treating population as a random factor. The factor range and its contrasts were tested against population, and the interactions of range and its contrasts with garden against garden:population. Garden was tested against the residuals.

	Height				Diameter			
Term	Df	SS	P	SS%	Df	SS	P	SS%
Rhizome mass	1	5719.49	0.000	3.26	1	417.86	0.000	3.32
Garden	1	43034.88	0.000	24.53	1	6409.04	0.000	50.89
Range, contrasts:	3	67752.28	0.000	38.62	3	1945.38	0.000	15.45
a) CN vs. JA/EU/US	1	67626.07	0.000	38.55	1	1882.22	0.000	14.94
b) JA vs. EU/US	1	19.23	0.703	0.01	1	54.69	0.010	0.43
c) EU vs. US	1	106.98	0.370	0.06	1	8.46	0.305	0.07
Population	124	16352.99	0.000	9.32	124	990.26	0.000	7.86
Garden:Range, contrasts:	3	411.8	0.150	0.23	3	476.22	0.000	3.78
a) Garden:CN vs. JA/EU/US	1	255.93	0.069	0.15	1	437.84	0.000	3.48
b) Garden:JA vs. EU/US	1	155.85	0.155	0.09	1	36.58	0.001	0.29
c) Garden:EU vs. US	1	0.02	0.987	0	1	1.8	0.462	0.01
Garden:Population	112	8509.91	0.000	4.85	112	369.05	0.071	2.93
Residuals	737	33657.72	NA	19.18	737	1986.83	NA	15.78

	Leaf size				Leaf biomass			
Term	Df	SS	P	SS%	Df	SS	P	SS%
Rhizome mass	1	138375.99	0.000	5.12	1	466.34	0.000	0.33
Garden	1	159086.49	0.000	5.88	1	110714.09	0.000	78.37
Range, contrasts:	3	701621.02	0.000	25.94	3	416.21	0.252	0.29
a) CN vs. JA/EU/US	1	693373.11	0.000	25.64	1	391.91	0.051	0.28
b) JA vs. EU/US	1	204.79	0.833	0.01	1	7.63	0.783	0.01
c) EU vs. US	1	8043.12	0.187	0.3	1	16.67	0.685	0.01
Population	124	566693.95	0.000	20.95	123	12369.59	0.000	8.76
Garden:Range, contrasts:	3	73847.37	0.000	2.73	3	65.81	0.606	0.05
a) Garden:CN vs. JA/EU/US	1	64817.01	0.000	2.4	1	8.18	0.633	0.01
b) Garden:JA vs. EU/US	1	482	0.655	0.02	1	36.85	0.311	0.03
c) Garden:EU vs. US	1	8548.36	0.062	0.32	1	20.79	0.446	0.01
Garden:Population	113	270986.06	0.000	10.02	113	4022.65	0.000	2.85
Residuals	734	793942.61	NA	29.36	706	13212.73	NA	9.35

Term	Stem biomass				Aboveground biomass			
	Df	SS	P	SS%	Df	SS	P	SS%
Rhizome mass	1	539.28	0.000	1.09	1	2785.24	0.000	0.73
Garden	1	31413	0.000	63.26	1	307806.35	0.000	80.78
Range, contrasts:	3	4989.35	0.000	10.05	3	1639.75	0.106	0.43
a) CN vs. JA/EU/US	1	4972.57	0.000	10.01	1	1611.08	0.015	0.42
b) JA vs. EU/US	1	15.99	0.545	0.03	1	16.72	0.801	0
c) EU vs. US	1	0.79	0.893	0	1	11.94	0.832	0
Population	123	5337.23	0.000	10.75	123	32327.96	0.000	8.48
Garden:Range, contrasts:	3	1805.01	0.000	3.63	3	1017.17	0.008	0.27
a) Garden:CN vs. JA/EU/US	1	1804.76	0.000	3.63	1	924.72	0.001	0.24
b) Garden:JA vs. EU/US	1	0.0525	0.951	0	1	79.88	0.326	0.02
c) Garden:EU vs. US	1	0.19	0.906	0	1	12.57	0.696	0
Garden:Population	113	1540.68	0.000	3.1	113	9284.38	0.000	2.44
Residuals	715	4035.92	NA	8.13	706	26184.4	NA	6.87

Term	No. ramets			
	Df	SS	P	SS%
Rhizome mass	1	533.96	0.000	2.12
Garden	1	1835.75	0.000	7.28
Range, contrasts:	3	12075.75	0.000	47.9
a) CN vs. JA/EU/US	1	12028.82	0.000	47.72
b) JA vs. EU/US	1	31.25	0.084	0.12
c) EU vs. US	1	15.69	0.219	0.06
Population	124	1277.45	0.288	5.07
Garden:Range, contrasts:	3	1920.24	0.000	7.62
a) Garden:CN vs. JA/EU/US	1	1751.84	0.000	6.95
b) Garden:JA vs. EU/US	1	168.38	0.000	0.67
c) Garden:EU vs. US	1	0.0135	0.957	0
Garden:Population	113	517.93	1.000	2.05
Residuals	735	7047.84	NA	27.96

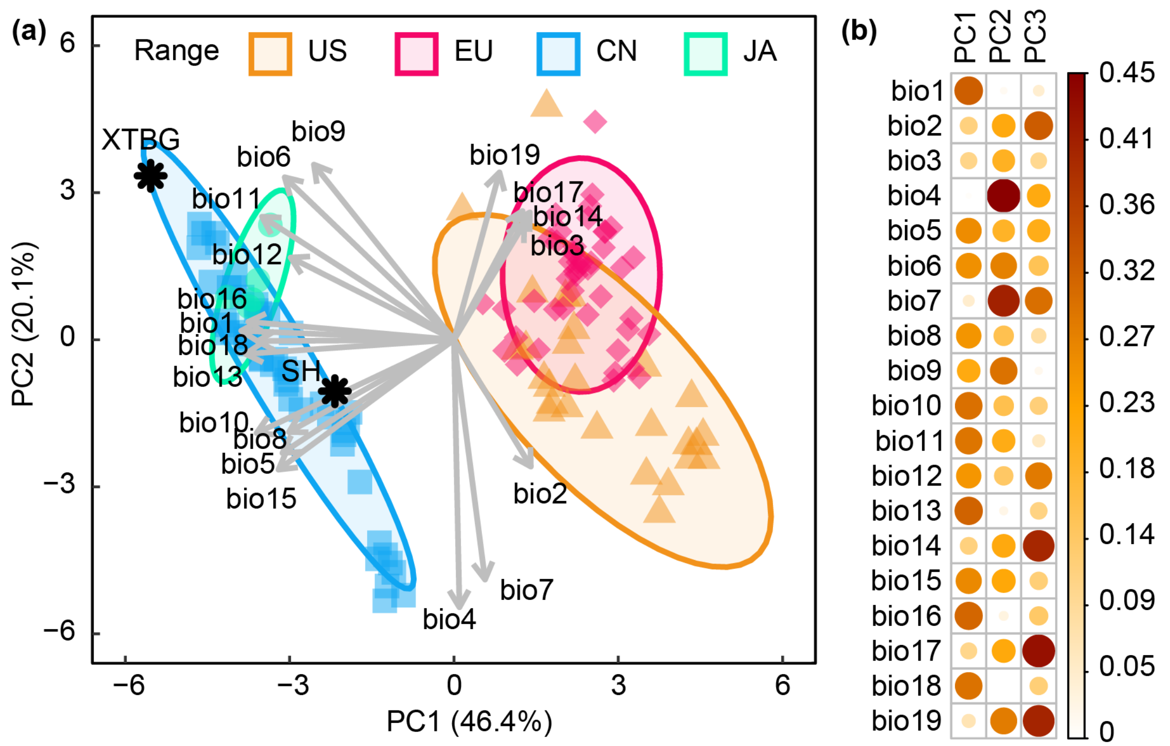

Fig. S1 (a) Biplot of principal component analysis of climate data (WorldClim bioclimatic variables) across *R. japonica* populations used in our study in the native (China and Japan) and introduced ranges (Europe and USA), and at the two common garden sites. Shaded polygons show 95% confidence ellipses for each range. BIO1 = Annual Mean Temperature; BIO2 = Mean Diurnal Range (Mean of monthly (max temp - min temp)); BIO3 = Isothermality (BIO2/BIO7) (×100); BIO4 = Temperature Seasonality (standard deviation ×100); BIO5 = Max Temperature of Warmest Month; BIO6 = Min Temperature of Coldest Month; BIO7 = Temperature Annual Range (BIO5-BIO6); BIO8 = Mean Temperature of Wettest Quarter; BIO9 = Mean Temperature of Driest Quarter; BIO10 = Mean Temperature of Warmest Quarter; BIO11 = Mean Temperature of Coldest Quarter; BIO12 = Annual Precipitation; BIO13 = Precipitation of Wettest Month; BIO14 = Precipitation of Driest Month; BIO15 = Precipitation Seasonality (Coefficient of Variation); BIO16 = Precipitation of Wettest Quarter; BIO17 = Precipitation of Driest Quarter; BIO18 = Precipitation of Warmest Quarter; BIO19 = Precipitation of Coldest Quarter. (b) Variable contributions to each PC. PC1 explained 46.4% of the climatic variation and was mainly associated with the annual mean temperature (bio1), the precipitation of the wettest month (bio13), the precipitation of the wettest quarter (bio16), the mean temperature of warmest quarter (bio10), the precipitation of the warmest quarter (bio18) and the mean temperature of the coldest quarter (bio11), all displaying negative loadings on the PC. PC2 explained 20.1% of the variation and was mainly associated with the temperature seasonality (bio4), the temperature annual range (bio7), the mean temperature of driest quarter (bio9), the precipitation of coldest quarter (bio19), the min temperature of coldest month (bio6) and the precipitation seasonality (bio15). The heat map scale indicates the loadings for each variable (i.e. the coefficients of the linear combination of the variables used to construct the PCA). The two PCs explained 66.5% of the total variation in climatic data across the four ranges.
